# Development and Validation of a UHPLC UV Method for the In-Process Control of Bosentan Monohydrate Synthesis

**DOI:** 10.1007/s10337-016-3124-y

**Published:** 2016-07-09

**Authors:** Marta Jatczak, Katarzyna Sidoryk, Magdalena Kossykowska, Wojciech Łuniewski, Joanna Zagrodzka, Elżbieta Lipiec-Abramska

**Affiliations:** 1 R&D Analytical Chemistry Department, Pharmaceutical Research Institute, 8 Rydygiera Street, 01-793 Warsaw, Poland; 2 Chemistry Department, Pharmaceutical Research Institute, 8 Rydygiera Street, 01-793 Warsaw, Poland

**Keywords:** UHPLC, Bosentan, In-process control, Drug substance, Validation

## Abstract

Bosentan monohydrate (4-*tert*-butyl-*N*-[6-(2-hydroxyethoxy)-5-(2-methoxyphenoxy)-2-(pyrimidin-2-yl) pyrimidin-4-yl]benzene-1-sulfonamide monohydrate) is a dual endothelin receptor antagonist (ERA) applied in the treatment of pulmonary arterial hypertension. To achieve effective process control of the bosentan monohydrate synthesis, it was necessary to develop a selective and not highly time-consuming method for ultra-high performance liquid chromatography (UHPLC). The method is characterized by adequate sensitivity, reproducibility and selectivity for the determination of bosentan monohydrate and related compounds from all synthetic stages. The UHPLC separation was carried out by reversed phase chromatography on the Acquity BEH C18 column (100 mm × 2.1 mm, 1.7 µm) with a mobile phase composed of solvent A (0.1 %, v/v, acetic acid in water) and solvent B (methanol), in the gradient mode at the flow rate of 0.4 mL min^−1^. Limits of detection and quantification for the compounds were ≤0.1 µg mL^−1^ and 0.3 µg mL^−1^, respectively. The linearity for all related compounds was investigated as in the range for the active pharmaceutical ingredient (API) and as in the range for the in-process control. The developed method was validated according to the current guidelines, proving the suitability of the method for its intended purpose.

## Introduction

Bosentan monohydrate, 4-*tert*-butyl-*N*-[6-(2-hydroxyethoxy)-5-(2-methoxyphenoxy)-2-(pyrimidin-2-yl)pyrimidin-4-yl] benzene-1-sulfonamide, is a selective mixed endothelin A and B receptor antagonist used in the treatment of pulmonary hypertension. Elevated endothelin concentrations are strongly correlated with the disease severity [2]. Bosentan decreases both pulmonary and systemic vascular resistance resulting in an increased cardiac output without increasing the heart rate. It is indicated for the treatment of pulmonary arterial hypertension (PAH) to improve exercise capacity and symptoms in patients with WHO functional class III. Some improvements have also been shown in patients with PAH WHO functional class II [3]. The drug is also indicated to reduce a number of new digital ulcers in patients with systemic sclerosis and ongoing digital ulcer disease [3–5].

**Scheme 1:**
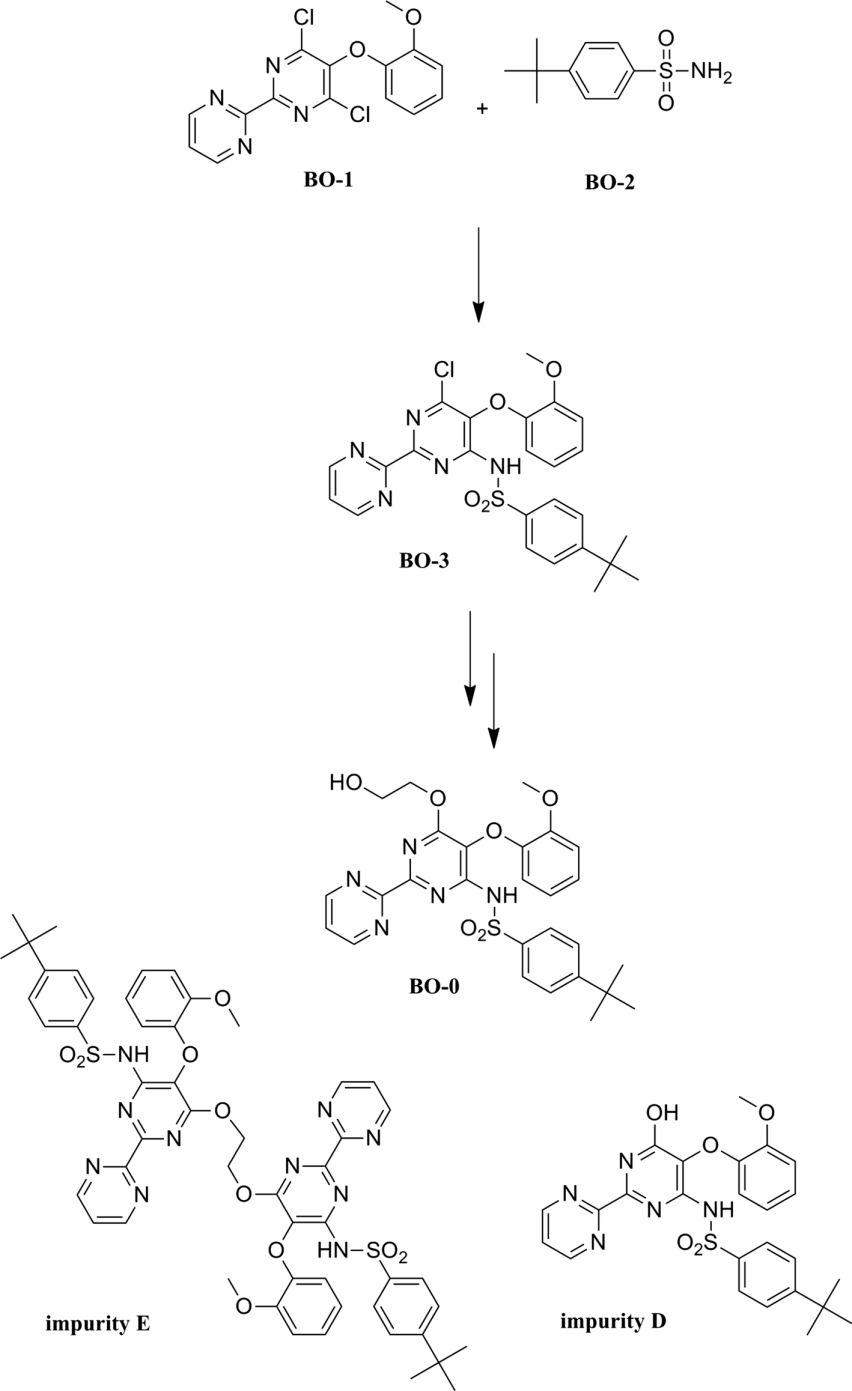
Bosentan monohydrate synthesis

Bosentan monohydrate can be obtained by the method described in the patent WO2014104904 A1 [6] shown in Scheme 1. In the first synthesis stage an intermediate compound—4-(*tert*-butyl)-*N*-(6-chloro-5-(2-methoxyphenoxy)-[2,2′-bipyrimidin-]-4-yl) benzenesulfonamide (BO-3) was obtained from the starting materials: 4,6-dichloro-5-(2-methoxyphenoxy)-2,2′-bipyridine (BO-1) and 4-(*tert*-butyl) benzenesulfonamide (BO-2). Bosentan monohydrate (BO-0) was obtained from BO-3 in the second synthesis stage. This two-step synthesis is also known as the first-generation process consisting of two consecutive replacements of chlorine atoms that exist in the starting dichloropirimidine: the first by *tert*-butylbenzenesulfonamide and the second by ethylene glycol. The method is accompanied by the formation of two known undesired by-products: “deshydroxybosentan impurity” (impurity D) and “dimer impurity” (impurity E). Literature suggested [7] that major improvement in the reduction of these impurity levels can be achieved by means of three consecutive crystalisations (two from methanol:isopropyl acetate and one from ethanol:water); however, no additional data were revealed. Therefore, it is necessary to find an analytical method suitable not only to monitor the reaction and levels of the obtained by-products, but also the levels of these impurities in the samples from any purification method employed to improve the chemical purity of the product.

The majority of analytical papers focus on the determination of bosentan monohydrate and its metabolites in human and animal plasma [8–13]. Some references deal with the analysis of bosentan monohydrate in drug formulations [14–18]. There are also a few papers referring to the determination of the impurities in the active pharmaceutical ingredients (API) of bosentan monohydrate and compounds generated during the degradation study [1, 19, 20]. Some papers deal with the analysis of the in-process control (IPC) of the API [21, 22]; however, there is no uniform requirement to follow the guidelines for the IPC methods.

There are only a few publications describing spectrophotometric methods for the estimation of bosentan monohydrate in pharmaceutical dosage forms [15–17]. The majority of analytical papers concern HPLC with UV and MS detection as the techniques used for the separation and determination of bosentan, its metabolites and degradation products [8–14, 18–22]. The analytical procedure was published in the authorized USP Pending Monograph and in the United States Pharmacopeia Convention. It recommends applying HPLC UV for the determination of bosentan monohydrate and related substances [20, 21].

The aim of this study was to develop a selective UHPLC UV method for the determination of the compounds of the bosentan monohydrate synthesis. Time of analysis (in literature) is about 40–55 min and the method is too time-consuming for the in-process control of the reaction which takes 4 h. What is more, some of the methods reported in literature are not sufficiently selective. The main goal of this study was to develop a selective and not highly time-consuming method for ultra-high performance liquid chromatography for the determination of the starting materials, intermediates and product of the bosentan monohydrate synthesis. One method for all the synthesis stages allowed to control the impurities at different levels and to find their sources. The method was validated and forced degradation studies were performed in accordance with the International Conference on Harmonization (ICH) guidelines to prove the specificity and suitability of the developed method [24].

## Experimental

### Reagents and Reference Standards

The working standards of bosentan monohydrate, intermediate and impurities D and E were produced in Pharmaceutical Research Institute (Warsaw, Poland). The structure of the compounds was confirmed by ^1^H NMR, ^13^C NMR, API-MS and elementary analysis. The standards potency was established on the basis of the organic impurities determination (UHPLC UV), residual solvents determination (GC FID), measurement of water content (Karl Fisher method) and determination of sulphated ash. The starting materials BO-1 and BO-2 were purchased from Credimate Trading Limited (Shanghai, China) and Atomax Chemicals Co (ShenZhen, China), respectively. Methanol of HPLC super gradient, acetic acid (≥99.5 %) and dimethyl sulfoxide (≥99.7 %) were purchased from POCh S.A (Gliwice, Poland). Demineralized water (≥18.0 Ω cm^−1^) from Barnstead system (USA) was used. The water solution of acetic acid was freshly prepared and filtered through a nylon, 0.22 µm pore size membrane (Bellefonte, PA, USA).

### Instrumentation

The Dionex UHPLC system (Thermo Scientific, Sunnyvale, CA, USA) was equipped with a DAD 3000 RS detector, an LPG 3400 quaternary pomp, a WSP 3000 TRS autosampler, a 100 µL syringe, a degasser and a column oven. Chromeleon 7.0 software was used for data recording and processing. The chromatographic separation was performed using the Acquity BEH C18 column 130 Å, 100 mm × 2.1 mm × 1.7 µm (Waters, Ireland). The column and the samples were thermostated at 30 and 25 °C, respectively. Separation was achieved using the mobile phase consisting of eluent A (0.1 %, v/v, acetic acid in water) and eluent B (methanol super gradient grade) with the flow rate of 0.4 mL min^−1^. The analysis was carried out under gradient conditions such as time (min)/A (v/v), B (v/v); *T*_0.01_/70:30, *T*_1.0_/40:60, *T*_7.0_/40:60, *T*_7.1_ */*5:95, *T*_11.5_/5:95, *T*_11.6_/70:30 and *T*_14.0_/70:30. The injection volume was 3 μL, and DMSO:MeOH (1:9) was used as the injection washing solution.

The detector working in the scan mode from 200 to 400 nm was used for the analysis of the forced degradation samples. The detector wavelength was set at 220 nm for the determination of related compounds in the bosentan monohydrate samples and at 270 nm for the assay determination.

### Preparation of the Sample and Standard Solutions

Stock solutions of bosentan monohydrate and related compounds (BO-1, BO-2, BO-3 and impurity D) were prepared in methanol at the concentration of 1 mg mL^−1^. The stock solution of impurity E that is sparingly soluble in methanol was prepared in chloroform and diluted with methanol (CHCl_3_:MeOH, *2:3*, *v/v*) at the concentration of 1 mg mL^−1^. The stock solution of bosentan spiked with BO-1, BO-2, impurity D and impurity E at 10.0 µg mL^−1^ (1.0 % concentration level) was used as the system suitability solution for the in–process control (SSS_1_). The stock solution of bosentan monohydrate spiked with all the impurities at 1.5 µg mL^−1^ (0.15 % concentration level) was used as the second system suitability solution (SSS_2_). The reference solution was prepared by diluting bosentan monohydrate to 1 µg mL^−1^ (0.10 % concentration level).

### Preparation of the In-Process Control Samples

In-process control solutions were prepared in two stages. A small quantity of the sample was taken from the reaction mixture and diluted with methanol to reach the concentration of 5 mg mL^−1^—solution I. The concentration was calculated for a product prepared with 100 % reaction yield. An appropriate volume of solution I was diluted to 1 mg mL^−1^ concentration with methanol—solution II. Solution II was filtered through a nylon 0.22 µm pore size membrane.

### Preparation of the Forced Degradation Studies

Stress testing was performed based on the ICH guidelines and available literature data [25–28]. The working standard of bosentan monohydrate was subjected to various forced degradation conditions to check the specificity and stability of the sample. Solutions of bosentan monohydrate at 10 mg mL^−1^ were prepared in stressing media in order to check its stability in different hydrolysis conditions. The solutions of bosentan monohydrate were refluxed with 2 M HCl (24 h), H_2_O (24 h), 1 M NaOH (8 h). Bosentan monohydrate was also oxidized using 30 % H_2_O_2_ at room temperature for 24 h. Photostability was checked by exposing to UV radiation using a SUNTEST CPS + light cabinet (Heraeus, Germany) at 300-800 nm (ID65 standard) radiation in two types of vials—glass and quartz. The sample in the glass container was stressed for 14 h (20160 kJ m^−2^) and the sample in the quartz container for 4 h (5760 kJ m^−2^). Test was carried out according to the ICH Q1B guideline in 25 ± 1 °C and 80 ± 2 % relative humidity for 24 h. Temperature stability of a bosentan monohydrate sample was checked by drying the sample at 60 and 100 °C for 24 h. Then the degradation samples were cooled down to room temperature, diluted to 1 mg mL^−1^ with diluents and mixed. For each condition a blank solution was prepared in the same manner as the stressed sample of bosentan monohydrate by mixing the stressing medium with diluents. Bosentan monohydrate before degradation was used as the control sample. The samples were withdrawn and filtered using PTFE 0.22 µm syringe filters (Chromafil, Germany) and analyzed by UHPLC UV.

## Results and Discussion

### UHPLC UV Method Development and Optimization

**Fig. 1:**
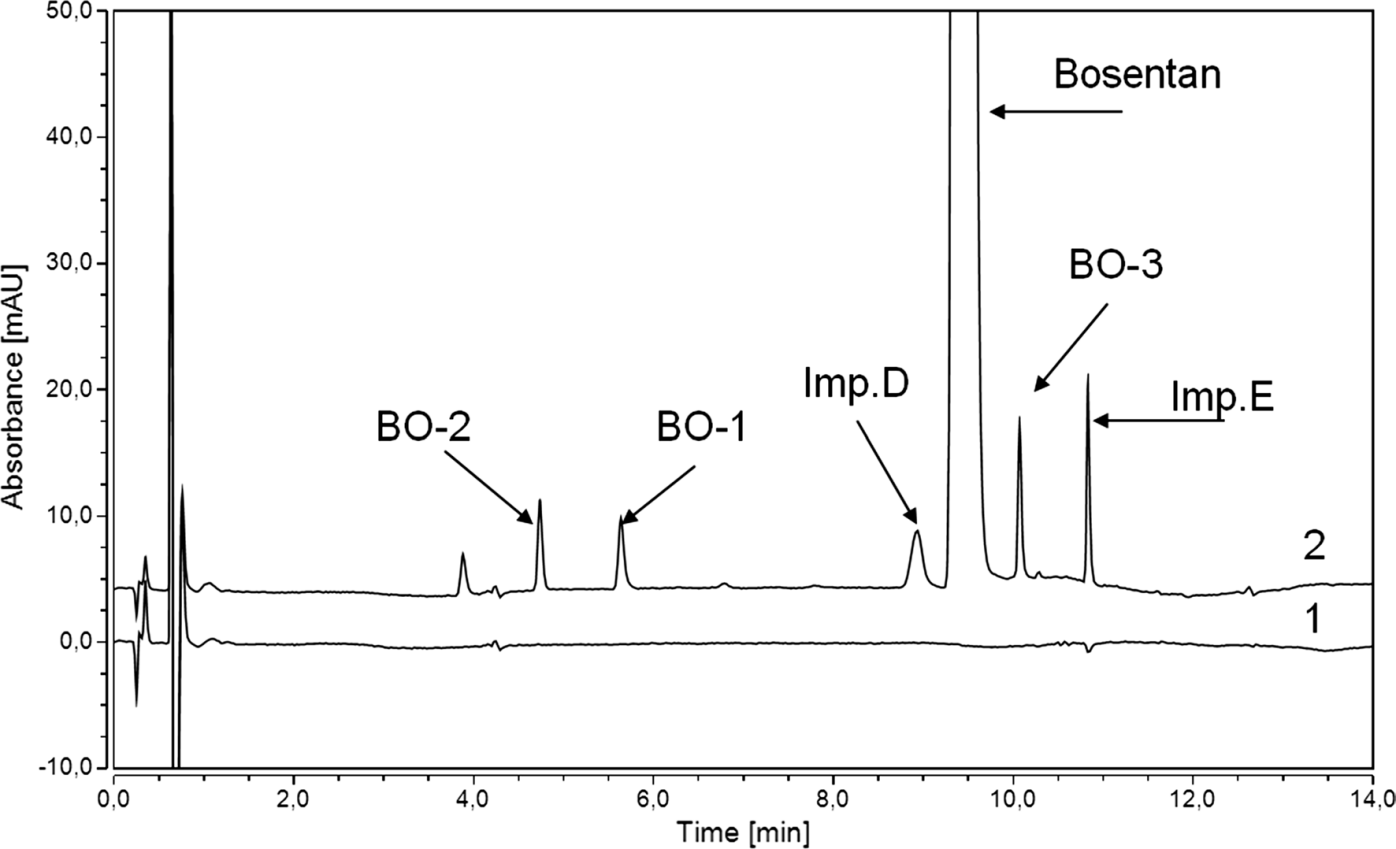
UHPLC UV chromatograms of the blank (*1*), system suitability solution (*2*)

It was necessary to develop a UHPLC UV method to efficiently control the reaction. To be used for efficient process control, the developed method should serve to separate all impurities in a short time. For this reason, a short column with small particle size was considered. The required selectivity and short method time were achieved on the Acquity BEH C18 column (100 mm × 2.1 mm × 1.7 µm). The column showed similar efficiency working with the flow rates between 0.2 and 0.5 mL min^−1^. Finally, because of the system limitations, the flow rate of 0.4 mL min^−1^ was selected. Bosentan monohydrate was found to exhibit two UV absorption maxima at approximately 220 and 270 nm. The 220 nm wavelength was selected for further method development of related substances determination in bosentan monohydrate while the 270 nm wavelength was selected for the assay determination method. The best peak symmetry and resolution were obtained for water with the addition of 0.1 %, v/v, acetic acid as mobile phase A. Methanol and acetonitrile were tested as organic mobile phase B. Methanol increased the intensity of the peaks, allowing to obtain a better signal-to-noise ratio and increased resolution between bosentan and impurity D. The high sensitivity of the method allowed for the determination of impurities at low concentrations. Gradient elution was used in the method to separate all related compounds. The required resolutions and symmetry for BO-1 and BO-2 as well as degradation products were achieved using gradient elution from 30 to 60 % of methanol. Because of the unsatisfactory resolution between impurity D and bosentan in gradient elution, isocratic elution was used from the first 2 min of the analysis. In the final step of the analysis, the share of the organic phase was increased to elute BO-3, impurity E and other degradation products. The chromatogram of the developed method is shown in Fig. 1. The solutions of bosentan and all impurities were stable at room temperature. It was not necessary to cool down the samples. The temperatures of the autosampler and the column were 25 and 30 °C, respectively. Temperature above 30 °C caused an increase in the resolution between BO-1 and BO-2, but at the same time decreased the resolution between impurity D and bosentan. The resolution between bosentan and impurity D and the RSD of the compounds area were chosen as critical parameters of the method. The method for the determination of the bosentan monohydrate and related compounds content was validated in accordance with the ICH guidelines [29, 30].

### Method Validation

Documents published by the International Conference on Harmonization (ICH) provide a framework for the method validation of the impurity assay in a drug substance and the assay of a drug substance, but there is no formal requirement to follow the guidelines for the method for the IPC assay [23, 24]. Control the starting materials purity requires a limited validation consisting of selectivity and LOD. The presented method for the assay control of the impurities in an API was validated in accordance with the ICH and consists of the selectivity and system suitability test, stability of the solutions, limit of detecton (LOD) and limit of quantification (LOQ), linearity in the range from the LOQ to 120 % of the concentration level, accuracy, precision and robustness. Bosentan was validated at the same levels as other known impurities to allow to calculate the assay of unknown impurities. The assay of unknown impurities was calculated in relation to bosentan. Moreover, the validation was extended by the elements of selectivity and system suitability test, LOQ for the starting materials and linearity range for the correct control of the synthesis process. The method was validated using the standard solution of bosentan monohydrate, starting materials (BO-1 and BO-2), intermediate product (BO-3) and impurities D, E, standard solutions.

### Selectivity and System Suitability Test

The selectivity and system suitability test (SST) of the method were determined separately for each stage (starting materials, in-process control and API chemical purity). Parameters such as repeatability (RSD of retention time and area), resolution and symmetry factors were determined and compared with the specification of the method. The resolution factors R_s_ must be higher than 1.5 for all pairs of peaks. For the starting materials, the selectivity and SST were examined with using BO-1 and BO-2 standard solutions (SS_BO-1_ and SS_BO-2_). The resolution between the peaks of BO-1 and BO-2 and their impurities was higher than acceptance criteria. The lowest resolution between BO-1 and its impurity with RRT 0.93 was 3.4. For BO-2 and its impurity with RRT 0.88 the lowest resolution was 6.1. The method was selective for the determination of the chemical purity of the starting materials. The results for the starting materials are presented in Table 1. For the in-process control, the selectivity and SST were examined using a system suitability solution (SSS_1_). In the chromatogram of the SSS_1,_ the resolution between peaks of bosentan and impurity D was the lowest and was equal 2.5, so the method was selective for the in-process control. The results are collected in Table 1. The selectivity and SST of the API chemical purity method was examined using system suitability solutions (SSS_2_). The results obtained during the system suitability test for the SSS_2_ proved that bosentan and its potential impurities could easily be separated from each other and the method was selective for the determination of the chemical purity in the API samples. The results are presented in Table 2.

**Table 1:** Results of validation method for substrates samples and in-process control

Parameters	Acceptance criteria	Purity of substrates						
		BO-1				BO-2		
		1^d^	2^d^	BO-1	3^d^	4^d^	BO-2	5^d^
Selectivity^a^								
R_s_	≥1.5	–	10.2	3.4	13.1	–	6.1	15.3
A_s_	0.8–1.5	1.2	1.3	1.3	1.0	1.2	1.2	1.1
LOD [µg mL^−1^], [%]	<<0.5, 0.05	–	–	0.1, 0.01	–	–	0.1, 0.01	–

Parameters	Acceptance criteria	In-process control					
		BO-2	BO-1	BO-3	Impurity D	Impurity E	Bosentan^c^
Selectivity^a^							
R_s_	≥1.5	8.9	18.0	4.8	2.5	11.7	–
A_s_	0.8–1.5	1.2	1.3	1.2	1.3	1.4	1.0
Range [%]		0.03–20	0.03–20	0.5–40	0.03–1.5	0.03–1.5	0.03–1.5
Linearity							
Slope		2.9331	2.4050	2.9971	3.1291	3.3795	3.0952
Standard deviation of slope		0.0101	0.0053	0.0241	0.0195	0.0066	0.0045
Intercept		0.0048	–0.0031	0.5659	0.0057	0.0090	–0.0005
Standard deviation of intercept		0.0830	0.0427	0.4085	0.0126	0.0039	0.0030
Correlation coefficient	>0.998	1.000	1.000	1.000	1.000	1.000	1.000
Standard deviation		0.2201	0.1223	0.8525	0.0269	0.0087	0.0066
*t*_cr_^b^	–	2.20	2.18	2.31	2.31	2.26	2.31
*t*_a_^b^	*t*_a_ > *t*_cr_	290.64	450.56	124.41	160.60	510.87	693.85
*t*_b_^b^	*t*_b_ < *t*_cr_	0.06	0.07	1.39	0.46	2.32	0.16

^a^Parameters of selectivity: resolution (*R*_S_) and symmetry factor (*A*_S_)

^b^Critical value of the Student *t* test: t(*α* = 0.95; *f* = *n* − 2)

^c^Parameter for unknown impurities were obtained for Bosentan

^d^1-imputiry of BO-1, Relative Retention Time—RRT = 0.75, 2-imputiry of BO-1, RRT = 0.93, 3-imputiry of BO-1, RRT = 1.35, 4-imputiry of BO-2, RRT = 0.88, 5-imputiry of BO-2, RRT = 1.35

**Table 2:** Results of validation method for determination of related compounds in API

Parameters	Acceptance criteria	API					
		BO-1	BO-2	BO-3	Impurity D	Impurity E	Bosentan^c^
Selectivity and SST^a^							
R_s_	≥1.5	18.6	9.2	5.4	2.6	13.2	-
A_s_	0.8–1.5	1.3	1.2	1.2	1.2	1.1	1.1
Range [%]		0.03-0.18	0.03-0.18	0.03-0.18	0.03-0.18	0.03-0.18	0.03-0.12
Linearity							
Slope		2.3958	3.0430	2.7145	3.1197	3.9697	3.0314
Standard deviation of slope		0.0102	0.0184	0.0123	0.0128	0.721	0.0032
Intercept		0.0007	0.0037	-0.0029	-0.0004	-0.0062	0.0002
Standard deviation of intercept		0.0012	0.0022	0.0014	0.0014	0.0078	0.0002
Correlation coefficient	>0.998	1.000	1.000	1.000	1.000	0.999	1.000
Standard deviation		0.0013	0.0024	0.0015	0.0016	0.0085	0.0002
*t*_cr_^b^	3.18	3.18	3.18	3.18	3.18	3.18	3.18
*t*_a_^b^	*t*_a_ > t_cr_	122.54	88.79	221.59	243.22	55.10	950.72
*t*_b_^b^	*t*_b_ < t_cr_	0.32	0.92	2.07	0.30	0.80	0.60
Response Factor		0.79	1.03	0.90	1.04	1.31	-
Accuracy							
Recovery from method I [%]	90–108	102.07	101.18	100.22	98.25	93.00	100.97
Recovery from method II [%]	90–108	99.86	100.06	97.37	98.33	100.41	100.77
Recovery from method III[%]	90–108	102.32	100.06	98.12	101.11	90.97	-
Repeatability							
RSD [%]	<1.0	0.86	0.36	0.51	0.63	0.49	0.69
Horrat’s test	HORRAT(r) value <1.3	0.16	0.06	0.08	0.10	0.08	0.12
Precision							
RSD [%]	<1.0	1.76	2.47	0.77	2.18	0.97	0.79
Horrat’s test	HORRAT(r) value <1.3	0.31	0.46	0.12	0.35	0.11	0.14
LOD [µg mL^-1^], [%]	<<0.5, 0.05	0.1, 0.01	0.1, 0.01	0.05, 0.005	0.1, 0.01	0.01, 0.001	0.1, 0.01
LOQ [µg mL^-1^], [%]	<0.5, 0.05	0.3, 0.03	0.3, 0.03	0.3, 0.03	0.3, 0.03	0.3, 0.03	0.3, 0.03
RSD for area [%]	<3.0	0.002	0.002	0.001	0.001	0.001	0.001

^a^Parameters of selectivity: resolution (*R*_S_) and symmetry factor (*A*_S_)

^b^Critical value of the Students *t* test: *t* (*α* = 0.95; *f* = *n* − 2)

^c^Parameter for unknown impurities were obtained for Bosentan

Selectivity tests proved that the symmetry factors for all peaks were in the range of 0.8 ≤ A_S_ ≤ 1.5.

Precision expressed as repeatability was calculated for the retention time and peak area and the RSD (relative standard deviation) was ≤3 % for the peaks under the 0.5 % concentration level. Moreover, the selectivity and SST of the API chemical purity method were examined using stress testing.

Degradation of bosentan monohydrate was carried out under acidic, alkaline, neutral, oxidative, thermolytic, photolytic and hygroscopic stress conditions. Studies of peak purity and assessment of mass balance for all the stressed samples showed the selectivity of the method. The peak match expresses similarity of the spectrum in the peak maximum and the spectra on the leading and trailing edges. Ideally, the spectra between the peak start and the peak end correspond to 100 % of the spectrum in the peak maximum, i.e., the peak match value is 1000. The match values showed no co-elution of the impurities and bosentan monohydrate. The resolution between the peaks was satisfactory and the mass balance values confirmed the validation acceptance criteria. The drug remained stable against photodegradation, hygroscopic and thermal degradation and oxidative hydrolysis. The main product of the degradation studies was impurity D. In neutral hydrolysis the assay of impurity D had increased twice. Significant degradation was observed in acidic hydrolysis (2 M HCl, 8 h, reflux). Bosentan was most degraded in alkaline hydrolysis (1 M NaOH, 8 h, reflux). The results are presented in Table 3.

**Table 3:** Results of forced degradation studies for bosentan monohydrate

Degradation conditions	Main peak purity	Degradation products		Sum of imp. [%]	Undegraded API [%]	Mass balance [%]
		RRT	Peak area ≥0.03 %			
Alkaline hydrolysis (1M NaOH, 8 h, reflux)	OK	0.64	0.09	9.90	90.17	100.07
		0.72	0.45			
		0.94 (Impurity D)	9.12			
		1.08 (BO-3)	0.04			
		1.15 (Impurity E)	0.15			
Acidic hydrolysis (2M HCl, 8 h, reflux)	OK	0.40	0.23	1.64	99.74	101.38
		0.45	0.10			
		0.54	0.07			
		0.94 (Impurity D)	1.07			
		1.08 (BO-3)	0.05			
		1.15 (Impurity E)	0.06			
Oxidative degradation (30 % H_2_O_2_, 8 h, RT)	OK	0.94 (Impurity D)	0.05	0.21	100.12	100.33
		1.08 (BO-3)	0.03			
		1.15 (Impurity E)	0.05			
Neutral hydrolysis (H_2_O, 24 h, reflux)	OK	0.94 (Impurity D)	0.12	0.34	99.86	100.20
		1.08 (BO-3)	0.06			
		1.15 (Impurity E)	0.07			
Photodegradation (Light, glass vial, 14 h, UV: 20160 kJ/m^2^)	OK	0.60	0.03	0.35	100.02	100.36
		0.95 (Impurity D)	0.11			
		1.07 (BO-3)	0.05			
		1.15 (Impurity E)	0.11			
Photodegradation (Light, quartz vial, 4 h, UV: 5760 kJ/m^2^)	OK	0.95 Impurity D)	0.11	0.33	100.58	100.91
		1.07 (BO-3)	0.05			
		1.15 (Impurity E)	0.11			
Photodegradation (Light, glass vial in aluminum foil, control sample)	OK	0.95 (Impurity D)	0.11	0.34	99.89	100.23
		1.07 (BO-3)	0.06			
		1.15 (Impurity E)	0.12			
Thermal degradation (100 °C, 24 h)	OK	0.95 (Impurity D)	0.12	0.40	95.29	95.69
		1.07 (BO-3)	0.06			
		1.14	0.03			
		1.15 (Impurity E)	0.12			
Hygroscopic degradation (25 °C, 80 % RH, 24h)	OK	0.95 (Impurity D)	0.11	0.34	99.66	100.00
		1.07 (BO-3)	0.05			
		1.15 (Impurity E)	0.12			
Control sample (not stressed)						
235-064-1 AAn/041/12	OK	0.94 (Impurity D)	0.11	0.32	99.91	100.23
		1.08 (BO-3)	0.05			
		1.15 (Impurity E)	0.11			
229-182-1 AAn/134/11	OK	0.94 (Impurity D)	0.05	0.18	99.82	-
		1.06 (BO-3)	0.04			
		1.15 (Impurity E)	0.06			

### Stability of the Solutions

A stability test was carried out on standard solutions (SS_BO-1_ and SS_BO-2)_, system suitability solution (SSS_1_, SSS_2_). The solutions were tested for stability within 24 h. Repeatability (retention times and peaks area), resolution and a symmetry factor were determined and compared with the specification of the method. The obtained results indicated that SS_BO-2_, SSS_1_ and SSS_2_ are stable for 24 h and SS_BO-1_ was stable for 3 h. During this time, no significant changes in the profile of the tested solutions were observed.

### Linearity

It was checked that the response of the detector is linear to the concentration of the analyte for all compounds. No less than five calibration standard solutions at different concentration levels (three analysis for each) were prepared and analyzed to receive sets of data points. The linearity was evaluated by a linear regression analysis to calculate the slope, *y*-intercept, and correlation coefficient (*R* ^2^). An additional restriction is that Student *t* test should be passed.

### Linearity for API

The calibration curve for unknown impurities was investigated in the concentration range 0.3–1.2 µg mL^−1^ (0.03–0.12 %), calculated in relation to bosentan and from 0.3 to 0.18 µg mL^−1^ (0.03–0.18 %) for all known impurities of the API (BO-1, BO-2, BO-3, impurity D and impurity E). Five solutions were used, each injected three times (*n* = 15). According to the results of the linearity test, the response factor for BO-1, BO-2, BO-3, impurity D and impurity E was determined. All results are presented in Table 2. All criteria were fulfilled.

### Response Factors

During the linearity studies, the response factor for every related substance was assessed. To assess the response factors, standards solutions containing the compounds at 3 or more different concentrations were prepared and analyzed. The response factors were calculated using a method based on comparing the slope of each compound’s regression line with the slope of bosentan monohydrate’s regression line. In the case of impurity E the response factor played an important role, because it was above the upper limit of 1.2—according to pharmacopeia [26]. The factor must be used as a correction factor when calculating the concentration with the use of the bosentan peak area. The response factors are presented as average values in Table 2.

### Linearity for the In-Process Control

In order to obtain effective process control, the linearity of the API method was checked in an extended range for all compounds. The calibration curve was investigated in the concentration range 0.3–200 µg mL^−1^ (0.03–20.0 %) for the starting materials (12 solutions for BO-1, *n* = 36 and 11 solutions for BO-2, *n* = 33). In the case of BO-3, the range was 0.5–40 % (5–400 µg mL^−1^) (13 solutions, *n* = 39). The calibration curve for known impurities was investigated in the concentration range 0.3–15 µg mL^−1^ (0.03–1.5 %), 8 solutions for impurity D, *n* = 24 and 9 solutions for impurity E, *n* = 27. The calibration curve for unknown impurities was calculated in relation to bosentan and was also extended. The curves for the in-process control were investigated in the concentration range 0.3–15 µg mL^−1^ (0.03–1.5 %), 8 solutions, *n* = 24. All results are presented in Table 1.

### Limits of Detection and Quantification

The limits of detection (LOD) and the limit of quantification (LOQ) are the same for the in-process control and for the API. The LOD and LOQ were estimated as signal-to-noise ratio. The values of signal-to-noise ratio were calculated by Chromeleon 7 software. Signal-to-noise ratio for the LOD and LOQ must be higher than 3.3 and not lower than 10.0, respectively. In accordance with the ICH, the reporting threshold should be lower than 0.05 % which corresponds to the 0.5 µg mL^−1^ concentration level for the analyzed compounds. The analytical method was sensitive enough as the LOQ was well below the reporting threshold. The precision for the LOQ level was below 3.0 %. The LOQ and LOD for all compounds were tested on the prepared three solutions at the concentrations levels close to the LOQ or LOD. All solutions were injected three times (*n* = 9). The results are shown in Table 2.

### Accuracy

There is no formal requirement to check the accuracy for the IPC method, so the accuracy studies were performed for the method only in the range used for the determination of the API purity.

The accuracy of the API method was separately determined for known related compounds and bosentan monohydrate. Model solutions were prepared at 3 concentration levels of the related substance—close to the LOQ, specification level and 120 % of the specification level, each injected three times (*n* = 27). A solution of bosentan monohydrate standard was used as matrix. The accuracy of the method for unknown impurities was determined by analysing the solutions of bosentan monohydrate standard at three different concentration levels (LOQ, 100 and 120 % of the acceptance level).

The accuracy of the method was investigated as the absolute recovery of sample solutions. Recovery studies for known compounds were performed using three calculation methods: applying linearity equation (method I), on the impurity standard independent point (method II) and on the bosentan standard independent point using a response factor (method III). The recoveries of unknown impurities (calculated on bosentan) were obtained using calculation methods I and II. Table 2 provides validation data results.

### Precision

The precision of the method for the API was studied for repeatability and intermediate precision. Differences between the results were tested by the RSD and statistical Horrat’s test.

The precision of the analytical procedure was tested by preparing 6 individual model solutions, analyzed in triplicate, *n* = 18. The RSD for all analyses was calculated for each impurity peak area. For the investigation of unknown impurities, sample solutions of bosentan monohydrate were prepared by diluting the stock solutions to their specification limit. Intermediate precision was carried out by two analysts using different instruments on different days. The results are presented in Table 2.

### Robustness

The robustness of the method is the ability to remain unaffected by small variations in the method parameters. The method robustness was evaluated by varying method parameters such as the percentage of the organic solvent (±2 %), percentage of acid in the mobile phase (0.05 and 0.15 %), column temperature (±3 °C) and flow rate (±0.01 ml/min), as well as wavelength (±2 nm). Method parameters were evaluated by changing one factor at a time. Changes in the chromatographic conditions slightly influenced the chromatographic parameters of the analysis. The resolution factors R_s_ were higher than 1.5 for all pairs of peaks. The symmetry factor A_s_ remained in the acceptance range for all compounds. The obtained results proved that the selectivity of the analysis did not change significantly under different conditions.

### In-Process Control

The presented method allowed to control intermediate compounds and impurities at different levels for all synthesis stages. The analysis of the substrate samples proved the absence of impurities which could lower the reaction yield. It was necessary to determine a UHPLC UV method to efficiently control the reaction. The results of the optimization of the synthesis process described in the patent WO 2014104904 A1. Short analysis time, selectivity and sensitivity of the method allowed for accurate monitoring of the reaction and changes in the level of impurities.

## Conclusions

The developed method for the in-process control of the bosentan monohydrate synthesis has significant advantages over other methods proposed in literature and official pharmacopoeial documents. The proposed method is short, sensitive, specific and selective enough to cope with the in-process samples. A wide scope of the in-process control allowed to find optimal reaction conditions and a purification method that would make the process efficient from the manufacturing perspective and help obtain a pharmaceutical grade product. Forced degradation studies and the extended range of the validation proved the usefulness of the developed method for its intended purpose.
